# Predicting ICU mortality in patients with abdominal aortic aneurysm: a nomogram based on MIMIC-IV and eICU-CRD

**DOI:** 10.7150/ijms.116265

**Published:** 2026-01-01

**Authors:** Mengwei He, Xiang Zhang, Weixue Huo, Jin Qu, Sen Wang, Zhaoxiang Zeng, Lushun Yuan, Rui Feng

**Affiliations:** Department of Vascular Surgery, Intervention Center, Shanghai General Hospital, Shanghai Jiao Tong University School of Medicine, Shanghai, 200080, People's Republic of China.

**Keywords:** abdominal aortic aneurysm, intensive care unit, mortality prediction, nomogram, risk stratification, clinical decision support

## Abstract

**Background:** Abdominal aortic aneurysm (AAA), characterized by pathological aortic dilation, carries high mortality in intensive care unit (ICU) settings. However, existing severity scores (e.g., SAPS III, SOFA) poorly capture AAA-specific mortality predictors. We aimed to develop a focused prognostic tool to improve short-term risk stratification in ICU-admitted AAA patients.

**Objective:** To develop and validate a machine learning-based nomogram model using the Medical Information Mart for Intensive Care IV (MIMIC-IV; 2008-2019) and the eICU Collaborative Research Database (eICU-CRD; 2014-2015) for early mortality prediction (≤7 days) in critically ill patients with AAA, addressing limitations of conventional ICU scoring systems by integrating AAA-specific predictors and ensuring generalizability through external validation.

**Methods:** Using two independent datasets from MIMIC-IV and eICU-CRD databases, we identified patients with AAA with complete ICU records and lab data within 24 hours of admission. Critical predictors were selected via a dual approach: least absolute shrinkage and selection operator (LASSO) regression to eliminate collinearity and support vector machine-recursive feature elimination (SVM-RFE) to rank feature importance. MIMIC-IV served as the training dataset, while eICU-CRD was used for external validation. A Cox regression-based nomogram was constructed using the training set and tested for 7-, 14-, and 28-day mortality prediction. Model performance was evaluated using area under the ROC curve (AUC), concordance index (C-index), calibration plots, and decision curve analysis.

**Results:** Six key variables independently predicted mortality including age, sepsis, blood urea nitrogen (BUN), antihypertensive drug use, average percutaneous arterial oxygen saturation (SpO_2_), and anion gap. The nomogram demonstrated optimal predictive accuracy for 7-day mortality (AUC: 0.730 [training] and 0.718 [validation]; C-indices: 0.717 and 0.731), with reduced performance for 14-day and 28-day outcomes. Calibration curves displayed strong agreement at both 7 and 14 days, and DCA indicated that the model provides significant clinical value. External validation in eICU-CRD mirrored these trends (7-day AUC: 0.723), supporting model generalizability.

**Conclusion:** This multicohort-derived nomogram provides a simple yet reliable tool to predict early mortality (≤7 days) in critically ill AAA patients, which may guide time-sensitive interventions in critical care settings.

## Introduction

Abdominal aortic aneurysm (AAA) is an abnormal dilation of the abdominal aorta^1^,^2^. The overall mortality rate following AAA rupture is 80%^3^, with approximately one-third of patients dying before reaching the hospital and 25-50% succumbing after undergoing surgery^4^-^6^. Given the high mortality rate, the intensive care unit (ICU) admission rate for AAA is significantly high^7^,^8^. Over the past three decades, the introduction of endovascular aneurysm repair (EVAR) and advancements in open surgical techniques have led to a reported decline in AAA mortality rates^9^. The mortality rate for elective open repair of AAA is 3-5%, whereas EVAR reduces the mortality rate to 0.5-2%^10^,^11^.However, a meta-analysis indicates that approximately 40% of AAA with complex anatomy are managed conservatively^3^,^12^,^13^. Due to the high ICU admission rate and mortality associated with AAA^7^,^8^, developing accurate and effective predictive models to assess prognosis of patients in ICU is particularly important for early identification of high-risk individuals and timely initiation of treatment.

Current scoring systems fail to bridge two critical gaps. First, conventional ICU tools such as OASIS, GCS, SAPS II, and SOFA^14^-^16^, are available to stratify patients based on the severity of their condition, but they prioritize generalized organ dysfunction over AAA-specific risks. Second, existing AAA-specific indices, such as Hardman Index (HI) and the Glasgow Aneurysm Score (GAS) 21,22, focused predominantly on surgical mortality in ruptured AAA populations 23, neglecting conservatively managed patients. While the Society for Vascular Surgery (SVS) guidelines provide several preoperative risk assessment indicators, including patient age, gender, smoking history, and type of surgery^5^. While these scoring systems are available to aid clinical decision-making in AAA, there is no conclusive evidence that they accurately predict survival outcomes for patients with AAA^24^,^25^.

This multicohort study aims to address these limitations by developing the first mortality prediction model tailored for ICU-admitted patients with AAA, leveraging the complementary strengths of the Medical Information Mart for Intensive Care IV (MIMIC-IV; 2008-2019) and the eICU Collaborative Research Database (eICU-CRD; 2014-2015)^29^. By systematically integrating demographic, laboratory, and therapeutic variables through machine learning algorithms, we seek to generate an actionable tool for early identification of high-risk patients, irrespective of surgical eligibility.

## Materials and methods

### Data source

The model was trained and internally validated using the MIMIC-IV database (version 2.2)^30^, followed by external verification using the eICU-CRD^31^. MIMIC-IV comprises critical care records from Beth Israel Deaconess Medical Center spanning 2008 to 2019, whereas eICU-CRD is a multicenter database that encompasses over 200,000 hospitalized ICU patients in the United States between 2014 and 2015. Reasons for ICU admission are severe illness, advanced age and multimorbidity. All personal identifiers have been removed and replaced with random codes, thereby exempting the study from patient consent and ethical approval.

### Study population and study design

**Figure 1:**
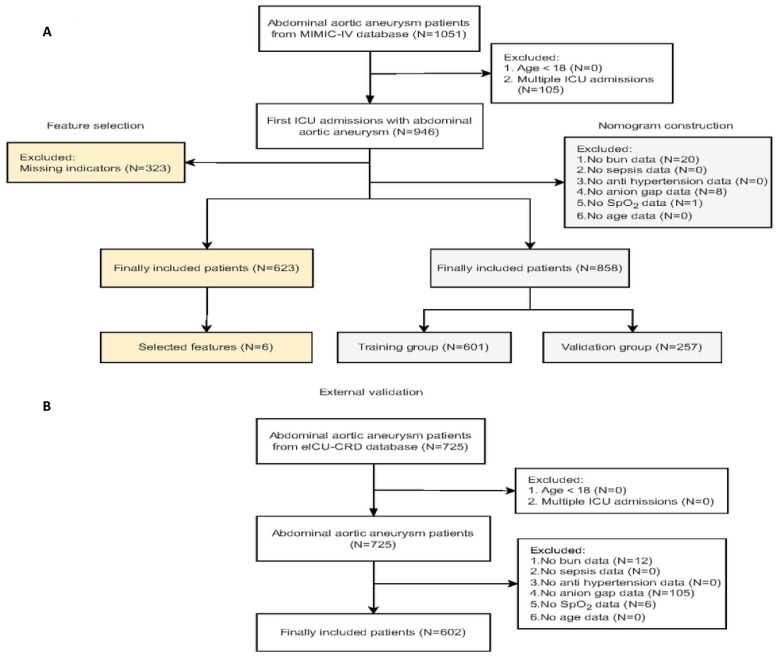
** Flow diagram of the patient selection in MIMIC IV and eICU-CRD.** Abbreviations: ICU: intensive care unit; MIMIC-IV: Medical Information Mart for Intensive Care; eICU-CRD: eICU Collaborative Research Database.

The eligibility criteria for this study included: 1) confirmed diagnosis of AAA based on ICD (international Classification of diseases)-9 and ICD-10 codes; 2) complete medical records from ICU admissions; and 3) availability of comprehensive data on laboratory tests and treatment interventions. Patients were excluded based on the following criteria: 1) age below 18 years; 2) multiple admissions during the study period; and 3) missing key indicators. We analyzed data on vital signs and laboratory results recorded within the first 24 hours of ICU admission, with the primary outcome being mortality during the ICU stay. Figure 1 illustrates the patient screening process and the study design.

### Data extraction

Data were extracted from the MIMIC-IV and eICU-CRD using PostgreSQL tools (version 17), Mengwei, He had the data use agreements. The data included demographic information (such as age, gender, and race), comorbidities (such as hypertension, diabetes, and coronary artery disease), laboratory test results (including complete blood counts, biochemical markers, and arterial blood gas analyses), and treatments (such as mechanical ventilation, dialysis, and surgical procedures).

### Statistical analyses

Descriptive baseline characteristics of the study population were expressed as median with interquartile range (IQR) for nonnormally distributed variables, mean with standard deviation (SD) for normally distributed variables, or percentages for dichotomous variables. Comparisons between groups were conducted using appropriate statistical tests, including student's *t-tests* for continuous variables and* chi-squared tests* for categorical variables. The significance level of *p* < 0.05 was considered statistically significant.

To simplify the model and identify significant predictive variables, we applied a stepwise feature selection process. First, Least Absolute Shrinkage and Selection Operator (LASSO) regression with L1 regularization was used to shrink less significant coefficients to zero^32^, while Support Vector Machine with Recursive Feature Elimination (SVM-RFE) identified features maximizing class boundaries. The intersection of results from both methods was taken to finalize key predictive variables. Subsequently, univariable Cox regression was performed to evaluate the association between these variables and ICU mortality in patients with AAA, followed by multivariable Cox regression to adjust for confounders and identify independent predictors. A nomogram was then created by assigning scores to each factor based on its impact on the outcome. After developing the nomogram, we calculated patient scores and stratified cases into low-risk and high-risk groups according to the median score. Differences in outcomes between these two groups were visualized using Kaplan-Meier (K-M) curves.

To assess the performance of the model, we employed several metrics, including the Time-dependent Area Under the Curve (AUC) and the Time-dependent Concordance Index (C-index) to evaluate its discriminative ability. Calibration curves were applied to evaluate the agreement between observed and predicted probabilities, reflecting the model's calibration and reliability. Additionally, Decision Curve Analysis (DCA) was conducted to assess the model's clinical utility by examining the balance between potential benefits and harms. Together, these methods offer a comprehensive evaluation of the model's performance, incorporating discrimination, calibration, and practical clinical relevance.

All analyses were primarily conducted using R software (version 4.2.1). The "glmnet" package was utilized for LASSO regression^32^, and the "rms" package was used to construct the nomogram for predicting mortality in ICU patients^33^. The "timeROC" and "pec" package were employed to obtain the time-dependent AUC and C-index respectively^34^,^35^. Calibration curves were generated using the "rms" package^36^, and DCA curves were constructed using the "rmda" package^37^.

## Results

### Patient selection and baseline characteristics

The MIMIC-IV and eICU-CRD contained 76,540 and 200,859 ICU admissions, respectively. Figure 1 depicts the patient selection process and the flowchart of the study cohort. A total of 623 patients with AAA from the MIMIC-IV database, with no missing indicators, were included for feature selection, resulting in the identification of six mortality-related features: blood urea nitrogen (BUN), sepsis, antihypertensive drug use, anion gap, average percutaneous arterial oxygen saturation (SpO_2_), and age. To increase the sample size, additional patients with missing data for other variables but complete information on six mortality-related features were included in the model development cohort. In total, 858 patients with AAA from the MIMIC-IV database were randomly split into training and validation cohorts in a 7:3 ratio. Additionally, we screened 601 patients with AAA from eICU-CRD for external validation. Table 1 outlines the demographic and clinical characteristics of all individuals included in the model, covering baseline features such as demographic factors, clinical characteristics, and available predictive variables. Among the 858 patients with AAA in the MIMIC-IV database, the in-hospital mortality rate was 42.89% (368 out of 858). In contrast, Additional table S2. presents data on mortality-related features for the 601 patients with AAA from the eICU-CRD, who had an in-hospital mortality rate of 4.16% (25 out of 601).

### Prognosis-associated feature selection

**Figure 2:**
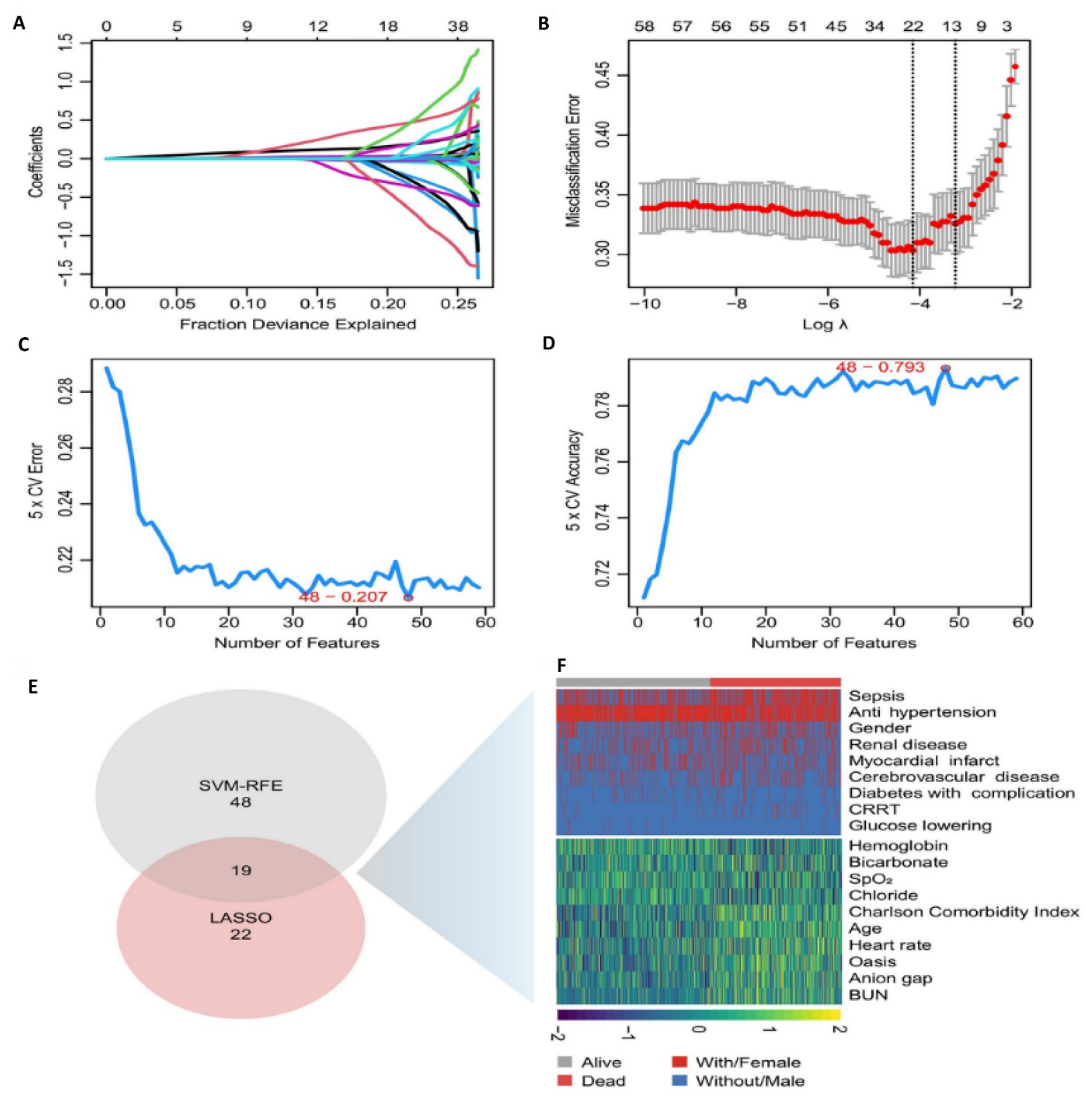
** The selection of feature variables. A.** Dotted vertical lines are drawn adopting the minimum rule and the 1 SE of the minimum rule at the suitable values log (λ), where factors are selected. **B.** 22 coefficients are included by LASSO coefficient profiles for clinical factors. **C.** Number of Features vs. 5-Fold Cross-Validation Error (SVM-RFE) Evaluates model performance with varying feature counts. The selected model minimizes cross-validation error. **D.** Number of Features vs. 5-Fold Cross-Validation Accuracy (SVM-RFE). Shows model accuracy improvement with increasing feature numbers. Best accuracy was achieved with 48 features. **E.** Feature Selection Summary. The intersection of the results from both algorithms resulted in 19 variables. **F.** Heatmap of Selected Features Visual representation of variable distribution between survival (alive) and mortality (dead) groups highlights key predictors used in the final model. Abbreviations: LASSO: least absolute shrinkage and selection operator. CV: Cross-Validation. SVM-RFE: support vector machine-recursive feature elimination. Bun: blood urea nitrogen. CRRT: continuous renal replacement therapy.

We used two different statistical methods to select feature variables. The LASSO regression algorithm identified 22 variables associated with ICU mortality in patients with AAA from an initial set of 59 variables (Figure 2A and B). Meanwhile, the SVM-RFE algorithm yielded 48 variables (Figure 2C and D). By taking the intersection of the results from both algorithms, we arrived at 19 significant variables: age, antihypertensive drug use, BUN, Charlson comorbidity index, anion gap, SpO_2_, renal disease, hemoglobin, oasis, sepsis, continuous renal replacement therapy, chloride, average heart rate, myocardial infarction, bicarbonate, antidiabetic drug, diabetes with complication, cerebrovascular disease (Figure 2E and F).

### Cox regression analysis

**Figure 3:**
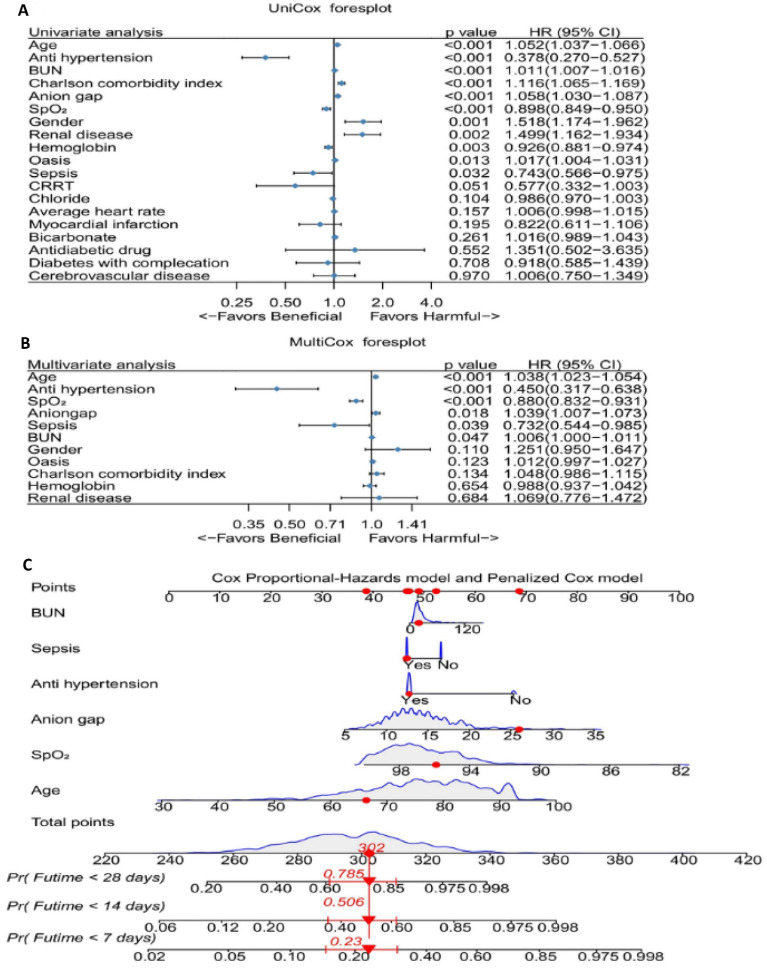
** The effect of selected factors on morality of patients with abdominal aneurysm. A.** UniCox regression analysis of the selected factors in the MIMIC IV database. **B.** UniCox regression analysis of the selected factors. **C.** The nomogram for prediction of hospital mortality among patients with abdominal aneurysm. Abbreviations: BUN: blood urea nitrogen. CRRT: continuous renal replacement therapy. Pr: Probability. Futime: follow-up time.

We performed univariate Cox regression analyses to determine the coefficients, hazard ratios (HR), and 95% confidence intervals (CI) for a total of 19 variables (Figure 3A). The analysis revealed 11 variables that were statistically significant. We then incorporated these 11 variables into a multivariable Cox regression model, which identified six clinical features significantly associated with mortality: BUN, sepsis, antihypertensive drug use, anion gap, average SpO_2_, and age (Figure 3B). Building on these results, we developed a nomogram (Figure 3C).

### Model performance

To evaluate the model's performance over time, we applied time-dependent AUC and time-dependent C-index (Additional file 1, Additional table S2), which allowed us to capture the dynamic changes in mortality risk for patients with AAA during the 28-day ICU follow-up. Over the 28-day period, both time-dependent C-index and AUC showed a gradual decline in predictive performance. Initially, the model demonstrated strong discrimination, with the highest values observed on Day 1, followed by a noticeable drop within the first 7 days. Between Days 7 and 14, both metrics stabilized but continued to decrease gradually. After Day 14, the decline became more pronounced, with the lowest values recorded beyond Day 20, indicating reduced long-term predictive capability. This trend suggests that the model is most effective for short-term mortality prediction, while its accuracy diminishes over time, likely due to evolving patient conditions and dynamic clinical factors in the ICU.

**Figure 4:**
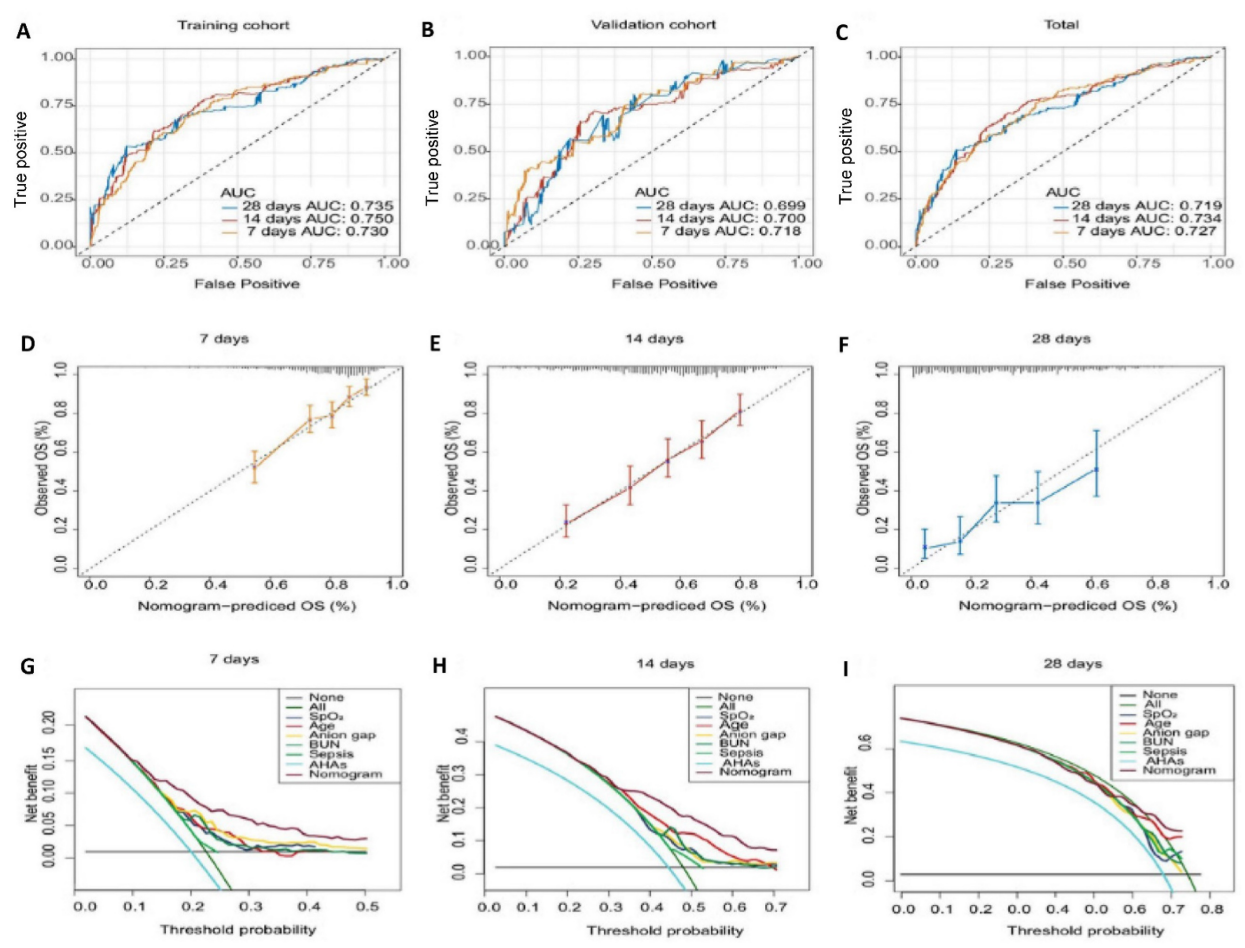
** Model performance evaluation. A-C.** The ROC curves of the nomogram for predicting in-hospital death risk of patients with AAA at 7, 14, and 28 days in the raining cohort (A), validation cohort (B), and total dataset (C). **D-F.** Calibration Curves of the nomogram for predicting in-hospital death risk of patients with AAA at day 7 (D), day 14 (E), day 28 (F). **G-I.** Decision curve analysis of the nomogram for predicting in-hospital death risk of patients with AAA at day 7 (G), day 14 (H), day 28 (I). *Abbreviations: ROC: receiver operating characteristic curve. AAA: abdominal aortic aneurysm. AHAs: antihypertensive agents. OS: overall survival.*

We observed significant changes in the time-dependent AUC and C-index at 7, 14, and 28 days, therefore, these intervals were used to define short-term, mid-term, and long-term predictions, with calibration and decision curves plotted to evaluate performance across these time frames. The results showed that the model achieved its best performance on Day 7, with a time-dependent AUC of 0.727 (Total), 0.730 (Training), and 0.718 (Validation), and a time-dependent C-index of 0.717 (Total), 0.713 (Training), and 0.731 (Validation). By Day 14, the predictive ability slightly declined, with a time-dependent AUC of 0.734 (Total), 0.750 (Training), and 0.700 (Validation), and a time-dependent C-index of 0.689 (Total), 0.696 (Training), and 0.684 (Validation). At Day 28, the model's performance further decreased, with a time-dependent AUC of 0.719 (Total), 0.735 (Training), and 0.699 (Validation), and a time-dependent C-index of 0.673 (Total), 0.682 (Training), and 0.676 (Validation). This analysis revealed that the model performs best in predicting short-term mortality, particularly within the first 7 days. However, its predictive ability gradually declines for medium-term (14-day) and long-term (28-day) outcomes. These findings highlight the necessity of dynamic monitoring and temporal adjustments in the ICU to improve long-term predictions (Figure 4A-C and Additional table S2).

Calibration curves indicated that the predicted probabilities of mortality closely aligned with the actual probabilities for both the 7-day and 14-day predictions, although consistency decreased at 28 days (Figure 4D-F).

The DCA for the nomogram revealed that the model demonstrated good clinical net benefit for predicting mortality at 7 days, 14 days, and 28 days, with the net benefit of the nomogram surpassing that of each individual feature (Figure 4G-I).

**Figure 5:**
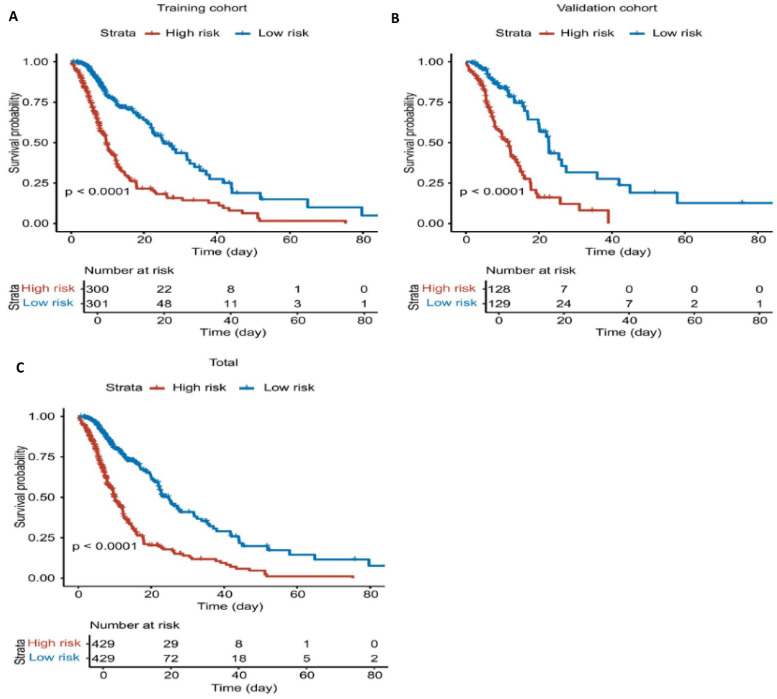
** Survival curves based on risk groups from the nomogram. A.** K-M plot of training cohort. **B.** K-M plot of validation cohort. **C.** K-M plot of total dataset.

Using this model, risk scores were calculated for all patients in the cohort (training and validation sets), and patients were stratified into high-risk and low-risk groups based on the median risk score. The results demonstrated that the model effectively distinguished between these groups, with the high-risk group showing significantly poorer outcomes compared to the low-risk group (Figure 5).

**Figure 6:**
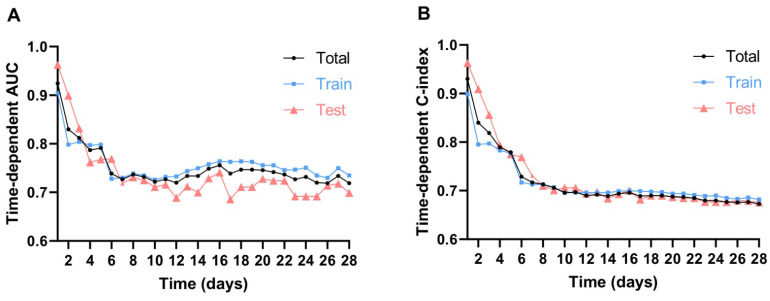
** Time-dependent AUC and C-index of eICU-CRD.** Abbreviations: AUC: area under the curve.

**Figure 7:**
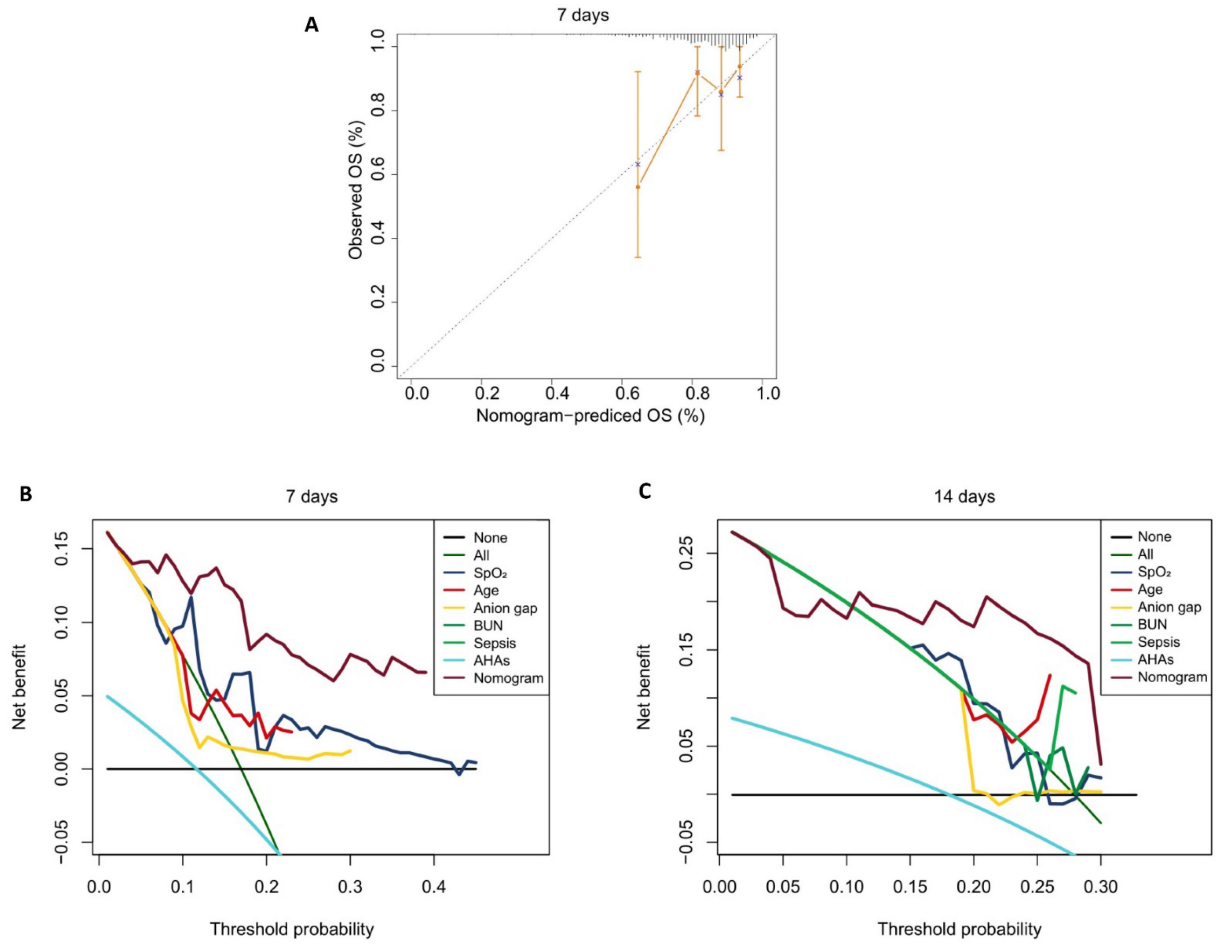
** The external model performance evaluation based on the eICU-CRD. A.** Calibration Curves of the model at 7-day. **B-C.** Decision Curve Analysis of the model at 7-day and 14-day.

In the external validation using the eICU-CRD, although the model's performance declined over the short term, it remained acceptable for 7 days, with the AUC decreasing from 0.935 on day 1 to 0.723 on day 7, and the C-index dropping from 0.804 to 0.548. After day 7, both AUC and C-index stabilized (Figure 6). This indicates the model maintains strong predictive accuracy for short-term mortality outcomes but exhibits diminished performance in forecasting longer-term endpoints, particularly beyond the 14-day timepoint. In the eICU-CRD cohort, the calibration curve demonstrated close alignment between the model's predicted 7-day mortality probabilities and observed outcomes (Figure 7A). DCA further revealed that the model provided superior risk stratification and higher clinical net benefit, supporting its utility in guiding 7-day and 14-day mortality risk stratification for patients with AAA in the ICU (Figure 7B and C).

**Figure 8:**
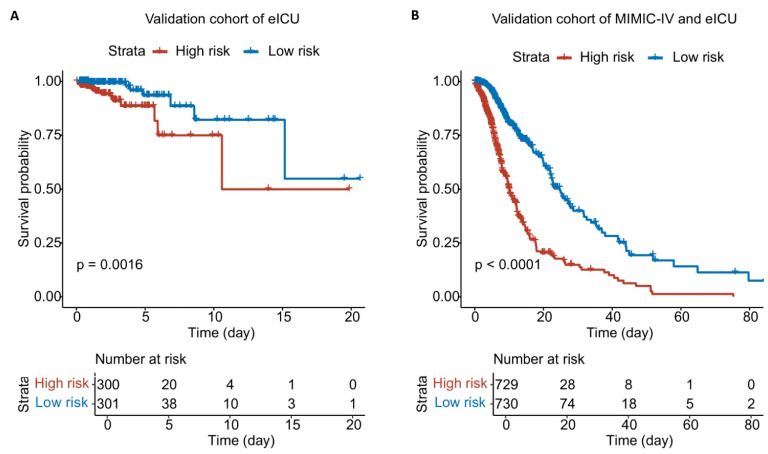
** K-M analysis of patients with AAA of two databases. A.** K-M plot of patients from eICU-CRD cohort. **B.** K-M plot of patients from eICU-CRD and MIMIC-IV population.

Using the nomogram, patients in the eICU-CRD cohort and the combined eICU-CRD/MIMIC-IV population were assigned risk scores and stratified into high- versus low-risk groups using median cutoffs. K-M analysis demonstrated good discriminative capacity of the nomogram across both the external validation cohort (eICU-CRD) and combined dataset, with the high-risk group exhibiting significantly poorer outcomes compared to the low-risk group (Figure 8).

## Discussion

Our study specifically examined the mortality risk for ICU patients with AAA, developed a novel predictive model based on real-time clinical data from ICU patients using stepwise comprehensive analyses, which can aid healthcare providers in better assessing the mortality risk for patients with AAA and optimizing treatment decisions.

Our findings indicated that BUN, sepsis, antihypertensive drug use, anion gap, average SpO_2_, and age have been identified as independent risk factors for predicting mortality in ICU patients with AAA. Based on these factors, we developed and validated an innovative nomogram model. The effects of most variables align with findings from previous studies in other domains. For instance, elevated BUN levels are linked to severe abdominal aortic calcification, raising cardiovascular risk and complicating clinical management in ICU patients with AAA 38,39. Despite antihypertensive medications not reducing AAA growth, they are crucial for managing blood pressure and lessening rupture risk, ultimately contributing to lower mortality rates 40-42. An increased anion gap generally indicates metabolic acidosis and is associated with higher mortality in AAA patients in the ICU 43-46. Additionally, low SpO₂ levels signal compromised respiratory function or inadequate oxygen supply; lower SpO₂ has been linked to increased mortality and severe complications after AAA repair 47-49. Age is also a key determinant of mortality risk, surgical outcomes, and treatment selection, with a 4% annual increase in mortality risk observed for patients over 65 years 50-52. Interestingly, the role of sepsis in our findings contrasts with existing literature. While sepsis is known to induce multiple organ dysfunction and heighten cardiovascular risk in AAA patients-significantly increasing the risks of aortic rupture, myocardial infarction, and cerebrovascular events 53-56. Our study found that the presence of sepsis served as a protective factor for ICU patients with AAA. To further explore this counterintuitive association, we conducted first performed landmark analyses to demonstrate time-dependent effect (Additional Figure 1) and a subgroup analysis (Additional Figure 2) stratified by illness severity using five scoring systems (APSIII, OASIS, SAPSII, SOFA, and Charlson Comorbidity Index). The results revealed a time-limited (strongest within 24hours) and severity-dependent pattern: in the most critically ill subgroup, especially in “very high”, sepsis was significantly associated with decreased mortality risk (OASIS: HR = 0.47; APSIII: HR = 0.57; SOFA: HR = 0.51; SAPSII: HR= 0.50). In contrast, sepsis showed either a neutral or elevated risk in lower severity subgroups—for example, the OASIS medium subgroup exhibited a hazard ratio of 1.50. These findings support the hypothesis that early recognition and intensive care in high-risk septic patients may yield improved outcomes. This unexpected result may stem from selection bias: both MIMIC-IV and eICU-CRD focus on critically ill patients, with those having sepsis likely identified as high-risk early on, leading to more aggressive interventions, such as early antibiotic administration and fluid resuscitation, which could lower mortality rates. Additionally, sepsis is well-studied with established management guidelines^57^,^58^, potentially resulting in better monitoring, early antibiotic therapy, fluid resuscitation and organ support for septic patients in the ICU. This aligns with modern sepsis care protocols, which emphasize rapid triage and resource prioritization. Conversely, patients with lower severity scores may receive less intensive management or derive less benefit from such interventions, contributing to the observed heterogeneity in outcomes. Our analysis highlights the need to interpret the impact of sepsis within the broader clinical context of baseline severity and care responsiveness. Further investigation into the impact of sepsis on this specific patient population is warranted^16^,^17^.

The predictive model has several advantages, including the incorporation of multiple clinical and demographic variables, allowing for individualized mortality risk assessment in ICU patients with AAA. It demonstrates strong predictive performance for short-term (7-day) mortality, supporting timely clinical decision-making. Therefore, its clinical utility is mainly limited to short-term prognostic assessment in ICU patients with AAA. Besides, the high mortality rate observed in the MIMIC-IV cohort reflects the critically ill nature of ICU patients with AAA, who were typically elderly and had multiple comorbidities. This focus enhances the model's relevance for ICU populations but limits its generalizability to non-ICU or elective surgical cohorts. Although ICU length of stay was not explicitly listed as a baseline characteristic in the tables, it was in fact incorporated into our analysis as the time variable in the Cox proportional hazards model. Moreover, we additionally compared ICU length of stay between patients stratified into high- and low-risk groups based on our model scores (Additional Figure 3). We found that high-risk patients had a shorter median ICU stay, which is consistent with higher early mortality. In contrast, low-risk patients exhibited longer ICU stays, likely reflecting prolonged survival and ongoing clinical management. These findings support the clinical utility of the model in early risk stratification and outcome interpretation. In addition, external validation shows that the model also exhibits stable performance in eICU-CRD, which enhances the reliability of the model. Another strength of our model is the superior performance compared with established ICU scoring systems, including APSIII, OASIS, SOFA, Charlson Comorbidity Index, and SAPSII. K-M survival curves demonstrated that the nomogram provided the clearest separation between high- and low-risk groups, whereas traditional scores showed weaker discrimination (Additional Figure 4). ROC curve analysis further confirmed higher AUC values for our model, particularly in predicting 7-day mortality (Additional Figure 5). Moreover, DCA indicated that the nomogram yielded greater net clinical benefit across a broad range of threshold probabilities, especially within the clinically relevant low-to-intermediate risk range (Additional Figure 6). Collectively, these findings underscore the enhanced discriminatory ability and clinical utility of the nomogram for ICU patients with AAA. This observation highlights a significant gap in risk prediction for this specific cohort, as these general scores were not designed to capture the unique pathophysiology and high mortality risk associated with AAA in critical care settings. Our novel nomogram, developed specifically for this population, successfully addressed this gap by providing statistically significant stratification, thereby emphasizing the necessity and clinical relevance of a severity predictive tool customized for ICU patients with AAA. However, the model has certain limitations. Firstly, the primary limitation of this study is the reliance on publicly available database data, without validation using in-house datasets, which may limit the generalizability of the model to different populations or clinical settings. Moreover, there is a significant difference in mortality rates between the external validation cohort from the eICU-CRD database (4.16%) and the MIMIC-IV cohort (42.89%). This substantial disparity may introduce bias and impact the mode's generalizability, as differences in patient populations, ICU admission criteria, and treatment protocols across databases could influence predictive performance. Future studies should consider adjusting for these variations or validating the model in more diverse and representative cohorts to enhance its robustness. Additionally, surgical data in the MIMIC-IV databases were inquired using standardized procedure codes, but these records may not fully capture operative details or long-term outcomes. As surgical status was not identified as a significant predictor in our analyses, the model is primarily applicable to short-term mortality risk assessment in ICU patients with AAA, rather than postoperative prognosis. Moreover, we only recorded indicators from the first day of ICU admission, which may not reflect subsequent disease progression. The limitation could explain why the model demonstrated good predictive capability within 7 days but gradually weakened for mid-term (14-day) and long-term (28-day) predictions. Another limitation is that although six factors were included in the model, their relative weights were not quantified, which may affect the interpretability and clinical applicability of the model. The time-dependent nature of ICU indicators highlights the necessity for further research to explore how these variables change at different time points after onset and their predictive value^59^. Notably, the absence of aneurysm-specific anatomical details (e.g. aneurysm diameter, classification) in the current datasets prevented clinically meaningful subgroup analyses. Incorporating these morphological parameters in future validation studies could significantly refine risk prediction models.

## Conclusion

We developed and validated nomograms based on six factors (BUN, sepsis, antihypertensive drug use, anion gap, SpO_2_, and age) to predict mortality in ICU patients with AAA. The model is simple, practical, and based on readily available clinical data, making it easy to apply to early risk assessment and clinical decision-making in ICU settings. It shows good discrimination and clinical utility at an early stage (7-day) for identifying high-risk patients who may benefit from closer monitoring and timely intervention. However, the predictive power of the model declined over time, suggesting that future research should focus on integrating dynamic clinical parameters to improve medium-and long-term predictions. Despite its limitations, this nomogram provides a valuable tool that can be used to support personalized care and optimize outcomes for critically ill patients with AAA. In the future, dynamic clinical parameters and external multicenter validation will be needed to improve the generalization ability and long-term prediction accuracy of the model.

## Supplementary Material

Supplementary figures and tables.

Supplementary file.

## Figures and Tables

**Table 1:** Baseline characteristics of 858 patients with AAA from the MIMIC-IV database.

	Total (N=858)	Validation (N=601)	Num of NA	Test (N=257)
Mortality rate	42.89% (N=368)	42.93% (N=258)		42.80% (N=110)
				
**Characteristics**				
Age	77.29 (63.10-91.48)	77.37 (63.02-91.72)		76.90 (62.9-90.9)
Female, No. (%)	277 (32.3)	190 (31.6)		87 (33.9)
Race, No. (%)				
White	647 (75.4)	464 (77.2)		183 (71.2)
Other	211 (24.6)	137 (22.8)		74 (28.8)
				
**Laboratory Indicators**				
Hematocrit (%)	35.9 (27.6-44.2)	38.55 (29.85-47.25)		37.80 (30.20-45.40)
Hemoglobin (g/dL)	12.7 (9.5-15.9)	12.7 (9.4-16.0)		12.6 (9.7-15.5)
Mean corpuscular hemoglobin (pg)	30.60 (27.70-33.5)	30.55 (27.45-33.65)		30.70 (31.00-36.40)
Mean corpuscular hemoglobin concentration (g/dL)	33.2 (31.2-35.2)	33.1 (31.0-35.2)		33.3 (31.9-35.2)
Mean corpuscular volume (fl)	92 (85-97)	92 (84-100)		92 (85-99)
Platelet (K/uL)	202.0 (100.0-302.0)	203.5 (102.5-304.5)	3	202.0 (104-300)
White blood cell (K/uL)	8.70 (4.10-13.5)	8.70 (4.10-13.3)		8.70 (3.8-13.6)
Creatinine (mg/dL)	1.10 (0.40-1.80)	1.10 (0.40-1.80)		1.10 (0.40-1.80)
Anion gap (mmol/L)	14.0 (9.0-19.0)	14.0 (9.0-19.0)		14.0 (10.0-14.0)
Red blood cell (M/uL)	3.51 (2.51-4.51)	3.55 (2.55-4.55)	4	3.44 (2.44-4.44)
Glucose (mg/dL)	128.0 (69.0-187.0)	127.0 (68.0-186.0)	1	129.0 (71.0-187)
Bicarbonate (mmol/L)	23.0 (18.0-28.0)	23.0 (18.0-28.0)		23.0 (18.0-28.0)
Bun (mg/dL)	21.0 (5.0-37.0)	21.0 (4.0-38.0)		20.0 (5.0-35.0)
Calcium (mg/dL)	8.30 (7.30-9.30)	8.30 (7.30-9.30)	40	8.30 (7.30-9.30)
Chloride (mmol/L)	105.0 (98.0-112.0)	105.0 (98.0-112.0)		105.0 (98.0-112.0)
Potassium (mmol/L)	4.20 (3.5-4.9)	4.30 (3.60-5.00)		4.20 (3.40-5.00)
International normalized ratio	1.10 (IQR=0)	1.10 (IQR=0)	12	1.10 (IQR=0)
Prothrombin time (second)	12.5 (9.5-15.5)	12.60 (9.60-15.60)	12	12.30 (9.30-15.30)
Partial thromboplastin time (s)	29.80 (21.80-37.80)	29.90 (22.90-36.90)	43	29.80 (20.80-38.80)
Sodium (mmol/L)	139.0 (135.0-143.0)	139.0 (134.0-143.0)	51	139.0 (134.0-144.0)
Mean glucose (mg/dL)	128.33 (86.33-170.33)	129.45 (87.45-171.45)	12	127.40 (87.40-167.40)
Serum creatinine baseline (mg/dL)	0.80 (0.47-1.13)	0.90 (0.56-1.24)		0.80 (0.40-1.20)
Serum creatinine min (mg/dL)	0.90 (0.40-1.40)	0.90 (0.40-1.40)		0.80 (0.30-1.30)
Serum creatinine max (mg/dL)	1.20 (0.20-2.20)	1.20 (0.20-2.20)	16	1.20 (0.20-2.20)
				
**ICU-score**				
OASIS	32 (20-44)	32 (19-44)		33 (20-46)
GCS	15 (3-15)	15 (3-15)		15 (3-15)
APS III	43 (19-67)	43 (18-68)	8	42 (19-65)
CCI	7 (4-10)	7 (4-10)		7 (4-10)
GCV	22.41 (9.41-35.41)	22.40 (8.40-36.40)	2	22.56 (8.56-36.56)
SOFA	4 (0-9)	4 (0-9)	8	4 (0-8)
SAPS II	38 (22-54)	38 (23-53)		38 (16-54)
				
**Vital Sign**				
Heart rate (beats/min)	79 (61-97)	80 (62-98)		80 (60-100)
Systolic blood pressure (mmHg)	116 (95-137)	116 (95-137)	6	115 (93-137)
Mean blood Pressure (mmHg)	76 (63-89)	76 (63-89)		76 (65-87)
Respiratory rate (breaths/min)	19 (15-23)	19 (15-23)		18 (14-22)
Temperature (℃)	36.73 (IQR=0)	36.72 (IQR=0)	31	36.73 (IQR=0)
SpO_2_ (%)	97.0 (94.2-99.8)	96.7 (93.9-99.5)		97.1 (94.7-99.5)
				
**Comorbidity**				
Myocardial infarct, No. (%)	227 (26.5)	147 (24.5)		80 (31.1)
Congestive heart failure, No. (%)	257 (30.0)	180 (30.0)		77 (30.0)
Cerebrovascular disease, No. (%)	158 (18.4)	112 (18.6)		46 (17.9)
Chronic pulmonary disease, No. (%)	321 (37.4)	223 (37.1)		98 (38.1)
Mild liver disease, No. (%)	61 (7.1)	40 (6.7)		21 (8.2)
Diabetes without complication, No. (%)	134 (15.6)	93 (15.5)		41 (16.0)
Diabetes with complication, No. (%)	57 (6.6)	43 (7.2)		14 (5.4)
Severe liver disease, No. (%)	12 (1.4)	8 (1.3)		4 (1.6)
Paraplegia, No. (%)	31 (3.6)	21 (3.5)		10 (3.9)
Renal disease, No. (%)	242 (28.2)	177 (29.5)		62 (25.3)
Sepsis, No. (%)	482 (56.2)	342 (56.9)		140 (54.5)
Chronic kidney disease, No. (%)	241 (28.1)	176 (29.3)		65 (25.3)
				
Treatments*				
Surgery performed, No. (%)	3 (0.3)	1 (0.2)		2 (0.8)
Continuous Renal Replacement Therapy, No. (%)	32 (3.7)	22 (3.7)		10 (3.9)
Antihypertensive drug use, No. (%)	754 (87.9)	535 (89.0)		219 (85.2)
Dialysis present, No. (%)	26 (3.0)	19 (3.2)		7 (2.7)
Glucose lowering, No. (%)	14 (1.6)	10 (1.7)		4 (1.6)
Ventilation, No. (%)	737 (85.9)	509 (84.7)		228 (88.7)

Abbreviations: N/No: number. NA: not available. Bun: blood urea nitrogen. OASIS: Oxford Acute Severity of Illness Score. GCS: Glasgow Coma Scale. APS III: Acute Physiology Score III. CCI: Charlson Comorbidity Index. GCV: Geriatric Comorbidity Validation Index. SOFA: Sequential Organ Failure Assessment. SAPS II: Simplified Acute Physiology Score II. * All listed treatments were administered during ICU hospitalization.
