# Supplementary material for: Predicting ICU mortality in patients with abdominal aortic aneurysm: a nomogram based on MIMIC-IV and eICU-CRD
Source: Int J Med Sci. 2026 Jan 1;23(1):146–60. doi: 10.7150/ijms.116265 (PMC12702021; doi:10.7150/ijms.116265)
Supplement: Supplementary file 1 — Supplementary figures and tables. [file ijmsv23p0146s1.pdf]

Additional Table 2. Key factors data of 601 patients with AAA from the eICU-CRD.

	Total (N=601)
Mortality rate	4.16%(N=25)
Factors	
Age	73.00(60.00-86.00)
Anion gap (mmol/L)	9.0(4.0-14.0)
Bun (mg/dL)	16.0(7.0-25.0)
SpO_2 (%)	96.1(93.7-98.5)
Sepsis, No. (%)	231(38.44%)
Antihypertensive drug use, No. (%)	277(46.09%)

Abbreviations: Bun: blood urea nitrogen.

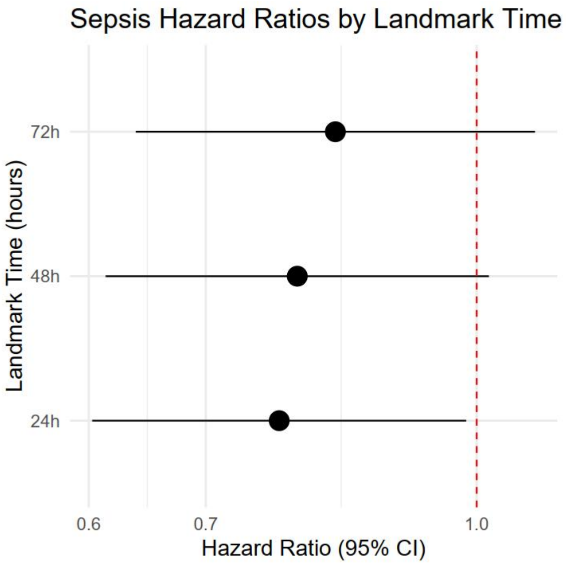

Additional Figure 1. Landmark analysis of time-varying effect of sepsis on ICU mortality.

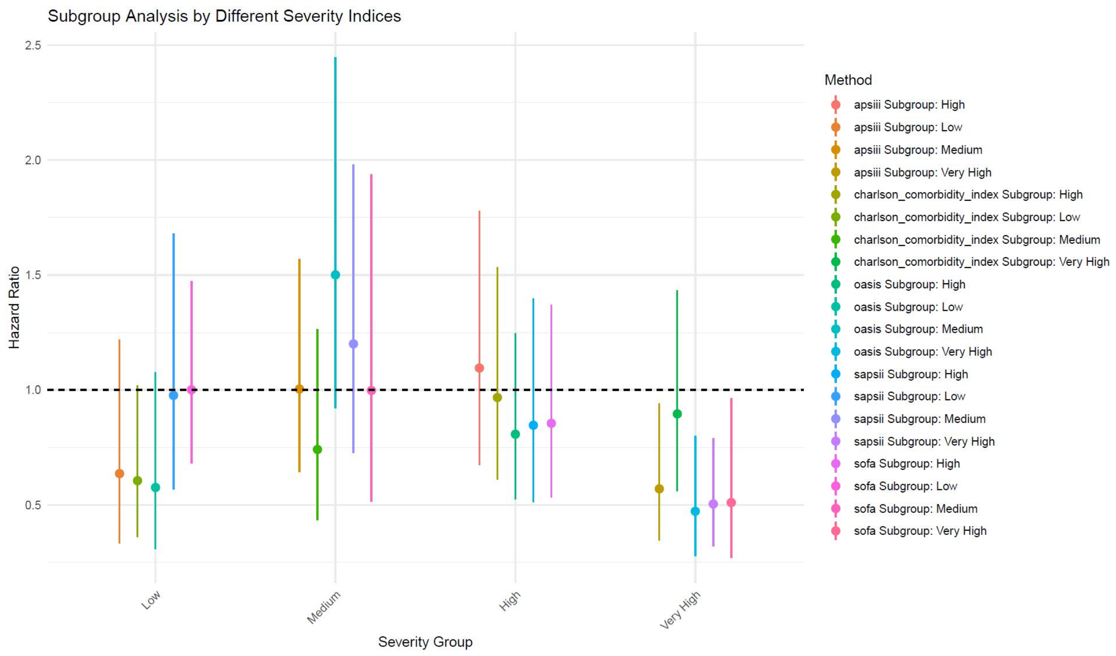

Additional Figure 2. Subgroup analysis of the association between sepsis and ICU mortality across different severity levels.

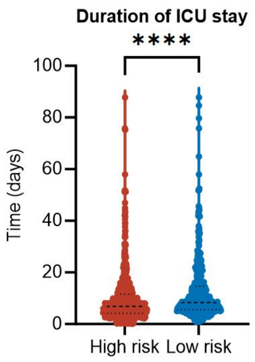

Additional Figure 3. Distribution of ICU length of stay in high- and low-risk groups.

****: p＜0.001

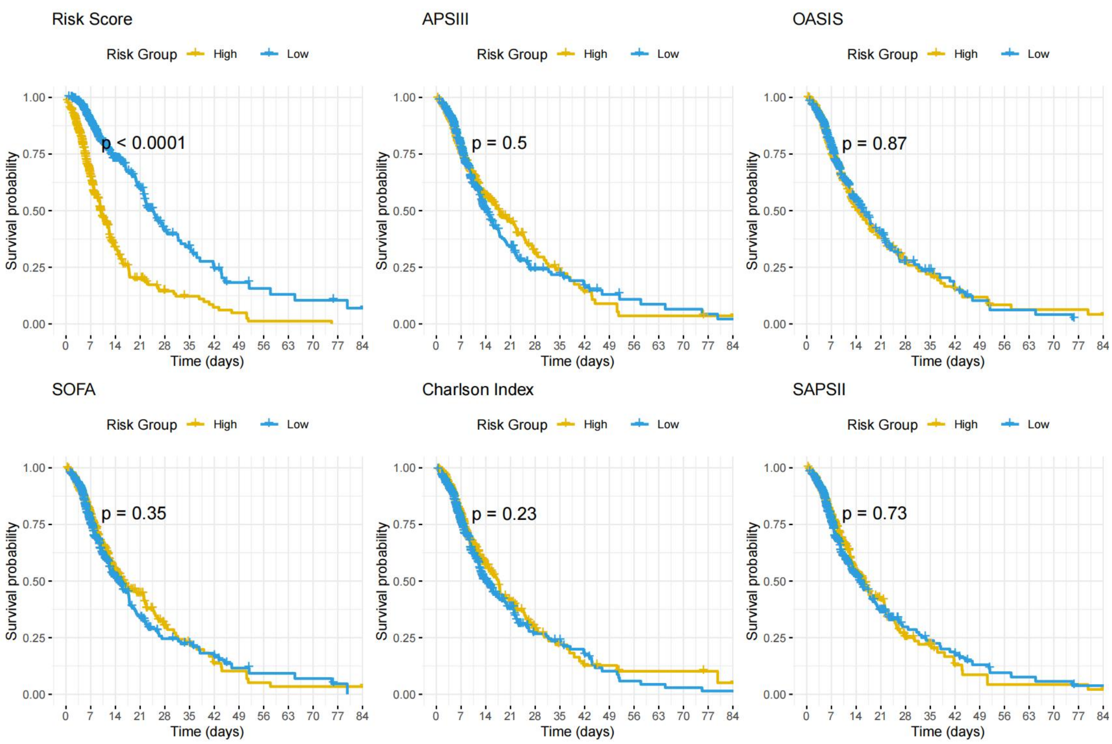

Additional Figure 4. K-M analysis Stratified by Nomogram Risk Score and Established ICU Scoring Systems.

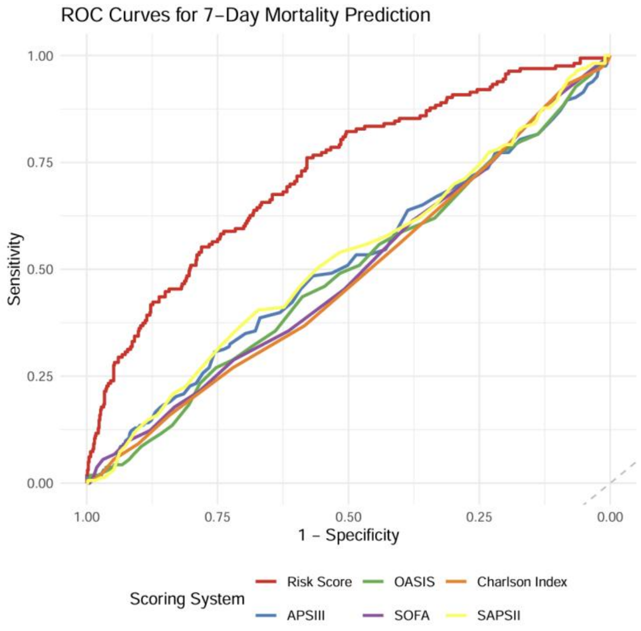

Additional Figure 5. ROC Curves Comparing the Nomogram with Established ICU Scoring Systems for 7-Day Mortality Prediction.

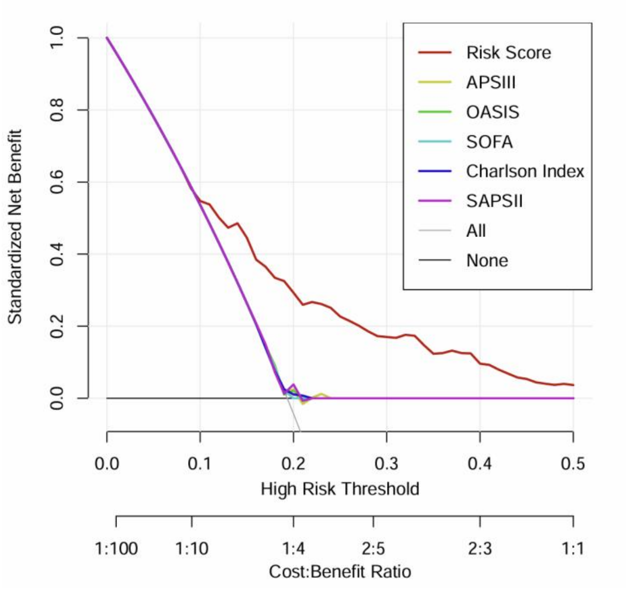

Additional Figure 6. Decision Curve Analysis of the Nomogram for 7-Day Mortality Prediction.
